# An assessment of requirements in investments, new technologies, and infrastructures to achieve the SDGs

**DOI:** 10.1186/s12302-022-00629-9

**Published:** 2022-07-01

**Authors:** Walter Leal Filho, Diogo Guedes Vidal, Chen Chen, Maria Petrova, Maria Alzira Pimenta Dinis, Peter Yang, Steven Rogers, Lorena Álvarez-Castañón, Ilija Djekic, Ayyoob Sharifi, Samara Neiva

**Affiliations:** 1 European School of Sustainability Science and Research, Hamburg University of Applied Sciences, Hamburg, Germany; 2 Department of Natural Sciences, Manchester Metropolitan University, Chester Street, Manchester, M1 5GD UK; 3 Centre for Functional Ecology, TERRA Associate Laboratory, Department of Life Sciences, University of Coimbra, Calçada Martim de Freitas, 3000-456 Coimbra, Portugal; 4 Faculty of Science and Technology, University Fernando Pessoa (UFP), Praça 9 de Abril 349, 4249-004 Porto, Portugal; 5 School of International Affairs and Public Administration and Institute of Marine Development, Ocean University of China, Qingdao, China; 6 The Earth Commons, Georgetown’s Institute for Environment and Sustainability, Georgetown University, 3700 O St NW, Washington, DC 20057 USA; 7 UFP Energy, Environment and Health Research Unit (FP-ENAS), University Fernando Pessoa (UFP), Praça 9 de Abril 349, 4249-004 Porto, Portugal; 8 Case Western Reserve University, 11112 Bellflower Road, Cleveland, OH 44106 USA; 9 School of Geography, Geology, and the Environment, Keele University, Keele, Staffordshire, ST5 5BG UK; 10 Social Science and Humanities, University of Guanajuato, Lascuráin de Retana No. 5, Col. Centro, 3600 Guanajuato, Gto. México; 11 Faculty of Agriculture, University of Belgrade, Nemanjina 6, Zemun, 11080 Belgrade, Republic of Serbia; 12 Graduate School of Humanities and Social Sciences, and Network for Education and Research on Peace and Sustainability, Hiroshima University, Higashi-Hiroshima, 739-8530 Japan; 13 Graduate Program in University Management, Federal University of Santa Catarina, Campus I-Roberto Sampaio Gonzaga, 274, Florianopilis, SC 88040-380 Brazil

**Keywords:** Investment challenges, Technological challenges, Infrastructural challenges, Achievement of UN SDGs, Bibliometric analysis, Case studies

## Abstract

**Background:**

The implementation of the Sustainable Development Goals (SDGs) requires much planning and the provision of resources, especially regarding the necessary investments, technologies and infrastructures needed. Yet, it is presently unclear how available these elements are, what gaps exist, what changes have taken place in terms of their availability since the adoption of the SDGs and what their requirements will be in the future. The knowledge gap has become even more concerning because of the impact of the COVID-19 pandemic. Using a bibliometric analysis, an assessment of the global progress of SDG implementation and requirements, identifying challenges through the development of a matrix, and a set of 11 case studies to triangulate the holistic analysis, an assessment of the global progress of the SDGs implementation and the impact of the COVID-19 pandemic on this process was carried out.

**Results:**

The findings suggest that the scope and width of resources limitation are currently undermining the implementation of the SDGs. Apart from the fact that the pace of progress has been insufficient, the potential of the SDGs in pursuing sustainability and improving life quality is not fully realised. This trend suggests that a substantial acceleration of the efforts is needed, especially for the five SDGs whose progress since 2015 has not been optimal, namely SDG2, SDG11, SDG13, SDG15, and SDG16, while SDG3, SDG7, SDG9, SDG14, and SDG17 show signs of progress. The case studies showed that different industries have dissimilar effects on achieving the SDGs, with the food sector correlating with 15 SDGs, as opposed to the energy sector correlating with 6 SDGs. Accordingly, the priority level assessment in terms of achieving the SDGs, points to the need to further advance the above-mentioned five SDGs, i.e., 2, 11, 13, 15 and 16.

**Conclusions:**

This study fills in a knowledge gap in respect of the current need for and availability of investments, new technologies, and infrastructures to allow countries to pursue the SDGs. It is suggested that this availability is rather limited in specific contexts. In respect of the needs to be addressed, these include resource-related constraints, limited technologies and infrastructures, affecting SDG2, SDG11, SDG13, SDG15, and SDG16, whose progress needs to be enhanced. Since the global progress in the process of implementation of the SDGs depends directly and indirectly on addressing the resource gaps, it is suggested that this topic be further investigated, so that the present imbalances in the three dimensions of sustainable development: the economic, social and environmental, be adequately addressed.

**Supplementary Information:**

The online version contains supplementary material available at 10.1186/s12302-022-00629-9.

## Introduction: the UN sustainable development goals

**Fig. 1:**
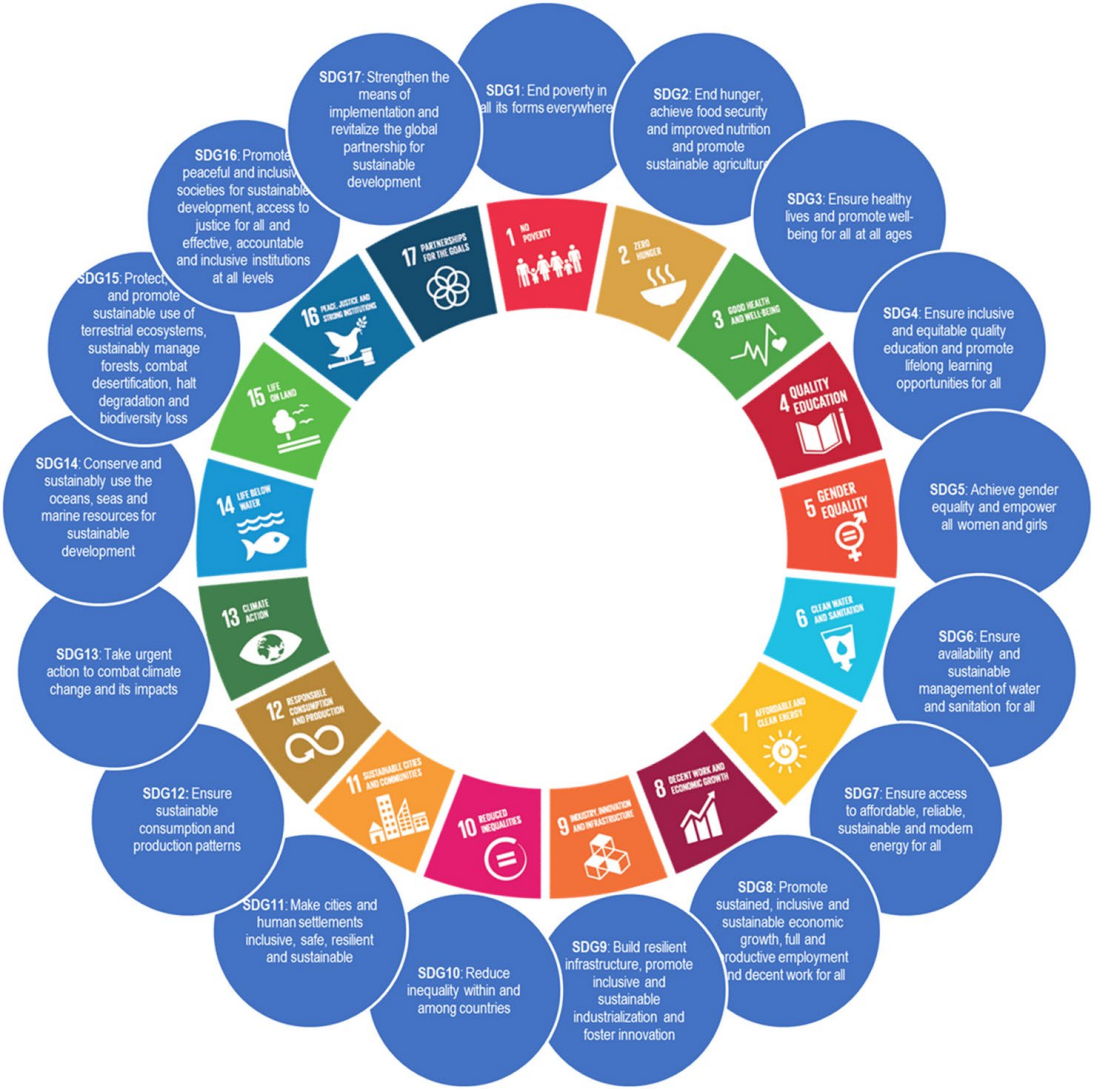
SDGs and their main purposes. Source: developed by the authors based on the UN’s Sustainable Development Goals

In 2015, the United Nations (UN) published the 2030 Agenda for Sustainable Development [1] in an attempt to solve key issues of the planet and achieve a positive future for the world. Through cross-national partnerships, the UN advocated for the fulfilment of the 17 Sustainable Development Goals (SDGs) and 169 targets concerning major aspects of people, planet, prosperity, and peace [2], as shown in Fig. 1. Massive scientific research has been conducted to discuss the SDGs [3]. However, two major questions have received much attention, namely: (i) what is the integrated meaning of the SDGs? (ii) what do the SDGs mean in different contexts?

In the process of exploring the first question, many researchers stated that the meaning and interactions of the different SDGs are part of an emerging area with a mass of knowledge gaps to be filled. Generally, researchers agreed that the 17 SDGs are indivisible, inclusive, and interactive [4–6]. Different goals and targets are closely linked, including environment and human health, policy and education, peace and business, among others [7]. Endeavours to reach one goal have the potential to influence the progress of achieving other goals, either positively or negatively [8]. Therefore, researchers from different fields are encouraged to participate in interdisciplinary collaborations to ensure that their behaviours not only contribute to a certain goal, but also generate positive impacts on other goals and targets. We argue that an assessment system of resources is necessary, which will balance its distribution among different SDGs, so that all goals and targets can be developed in an integrated way.

To answer the second question, various discussions have emerged in different regions, disciplines, and industries. There are several signs of progress and priorities for the implementation of the SDGs in different geographical regions [9–11]. Exercises to achieve SDGs by both developed and developing countries have offered valuable experiences to the rest of the world [12–17]. Different countries have diverse distribution strategies for SDGs investments, technologies, and infrastructures, based on their own needs, interests and abilities. These differences lead to different levels of contribution to the SDGs, as well as geographical limitations and unbalances [18]. Therefore, an assessment of requirements for different countries might benefit the appropriate distribution of resources.

Researchers from different disciplines have presented their diverse understandings of SDGs [19], where the specific focus has been on the fields of economy and environment. Economic cooperation such as international trade, integrated economic growth [20–22], and their interactions with other disciplines [23] are recognised as positive contributions to achieving the SDGs. Key environmental issues such as resource policy and climate actions [24], as well as their nexus relationship [25, 26], have been widely discussed. Other disciplines, including citizen sciences [27, 28], sustainability sciences [7, 29], spatial sciences [30] and policy sciences [31], among many others, also raised discussions on what SDGs mean to each discipline. Arguably, all the participated disciplines added not only values, but also requirements of resources for SDG development, yet failed to give appropriate considerations to how to assess the need for resources.

Different industries discussed the SDGs from their perspectives. Basic issues such as ocean management [32–34], agriculture protection [35, 36], sustainability education [37, 38], and their interactions with SDGs have been deeply explored. Perhaps the investments, technologies, and infrastructures to be applied within the different industries will inevitably generate various impacts such as displacement, resettlement, and other social impacts [39, 40]. To avoid negative impacts on the progress of SDGs implementation, good planning and an appropriate provision of resources among different industries are necessary.

It is clearly shown that the SDGs have raised interest in discussing their integrated meaning for various regions, different disciplines, and industries. The necessity of worldwide partnerships and cooperation has been widely valued. However, the implementation of the SDGs has met various difficulties, including poor planning of resource provision, which will be discussed below.

## Challenges associated with implementing the SDGs

There is an emerging body of work recording the implementation of the SDGs at a global level, but also more commonly at the national level [6]. The goals are being implemented across a spectrum of governing bodies, institutions, groups, companies, and individuals [41]. Records of implementation include policy, frameworks, reports, and academic outputs. Despite having 193 signatories, only 66 countries had started implementing the SDGs in the early phases of the UN’s proposed implementation period and have reported their progress via the Voluntary National Reviews [42]. Allen et al. [43] account that much of the implementation to date has been limited, with several reports outlining the already ongoing activities being linked to the SDGs, rather than new activities being informed by them. Alongside reports and reflections on activities aligning with and to the SDGs [44–46], there has been parallel commentary concerned with the progress of implementations [43], and indeed the feasibility of such implementations [47].

A major factor perceived to be a driver behind difficulties in implementing the SDGs is a lack of regular reviews [43], a general absence of collective, holistic, and linked actions [48, 49], and the absence of certain targets to reduce pressures. This means that the implementations may address the symptoms and not the causes [47]. Some authors, such as Amos and Lydgate [50], argue that one of the limitations of SDG implementation is the understanding of the frameworks involved. The ability to distinguish between goals that are related to the process and goals related to results is not certain. Some groups/individuals have an absence of transformative power, and for some decision-makers and stakeholders, the economic pillar of sustainability has gained more attention than the social and environmental pillar. This means that the action plans created do not reach their maximum potential, by not taking into account that the three pillars act in a comprehensive and integrated manner.

Several points that hinder the implementation of the SDGs can be found when analysing the literature and the studies of different authors, such as the lack of a process that is intersectoral to instigate the coherence of political plans. Due to its great conceptual multiplicity, the vast majority of the goals created cannot be satisfactorily transposed into facts that are measurable [51]. On the other side, Morton et al. [52] show that it is necessary to analyse whether the government officials have the necessary skills for the implementation of the SDGs. This is because in several cases it can be shown that the negative impacts of the implementation attempts occur because of inadequate management.

In order for the SDGs to be implemented, it is important to understand the correlation among the 17 goals, which is one of the points that can hinder their implementation due to the possibility that they may have different assignments [53, 54]. It is also important to highlight that when dealing with policies aimed at exploring the existing correlation between the SDGs, some interactions can develop in real-time, while others can present significant delays [51]. According to Amos and Lydgate [50], despite having been fulfilled as a global objective to ensure that all regions of the globe achieve economic growth, social development, and environmental preservation, the factual structures that contribute to the monitoring of goals and their development, especially those aimed at the SDGs interconnections, do not explicitly deal with the tension between the goods that are considered as global audiences and the quantification of the SDGs on national scales.

Another question related to the implementation of the SDGs was raised by Almeida et al. [55], when seeking to understand how to find ways to incorporate the links and the trade-offs, concerning the process of formulating strategic processes and planning involving all 17 SDGs. They observed that, in certain situations, existing compensations cannot be significantly mediated in an appropriate and recommended way. Breuer et al. [51] also emphasise that some of the goals established by the SDGs have generic relationships on a global scale, and thus can be better implemented in different contexts and regions. However, some of the goals were created to meet a specific need in a given geographical context.

Much of the literature regarding the SDGs implementation focuses on policy, frameworks [41], and academic or speculative aspects. What is less often reported are the technological, logistical, and resource-related constraints. These limitations are particularly interesting, as many of the technologies relying on logistics and resources are in development and their intrinsic impact is still being tested. Some of these promising technologies have been and are being tested, but often in isolated “pilot studies”, which would benefit from global information sharing and development, e.g., technology that removes arsenic from water [56]; high potential, but not yet mainstream, waste-to-energy technologies [57]; or bio-based technologies limited by the availability of biomass [58]. An underpinning technological and societal movement critical to the implementation of many of the SDGs is the transition away from fossil fuels. Neofytou et al. [59] highlight the thousands of publications that mention this energy transition and ultimately indicate that countries in the global South are less prepared for this transition. A good example of resource availability acting as a limitation to the energy transition is the availability of lithium, a major component of lithium-ion batteries. Tabelin et al. [60] review the demand and supply of lithium and the various mechanisms for its extraction and report that the extraction of lithium over the next 5 years will lag behind its demand.

## Methods

**Fig. 2:**
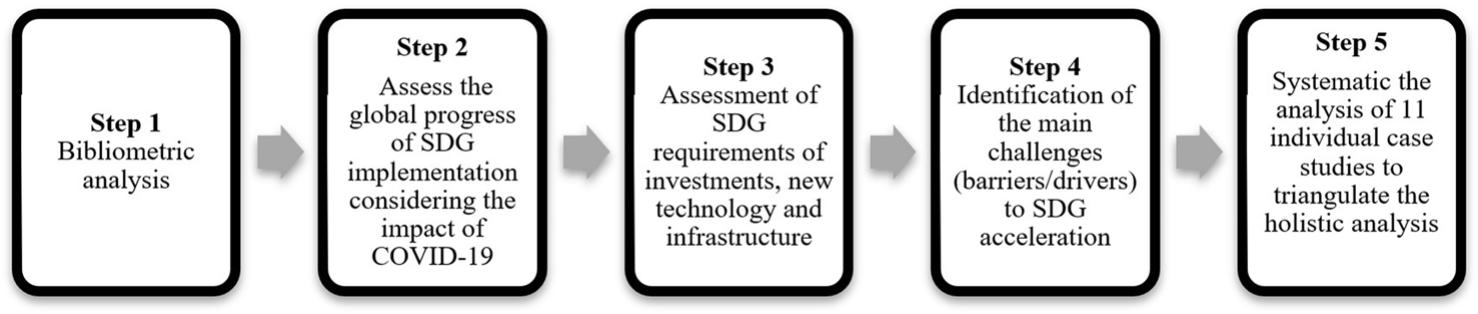
Steps of the research. Source: developed by the authors

This study aimed to identify and assess how governments, non-governmental organisations, and universities perceive, promote, and manage matters related to achieving the sustainable development goals (SDGs) and is based on the fifth main methodological steps described in Fig. 2.

**Fig. 3:**
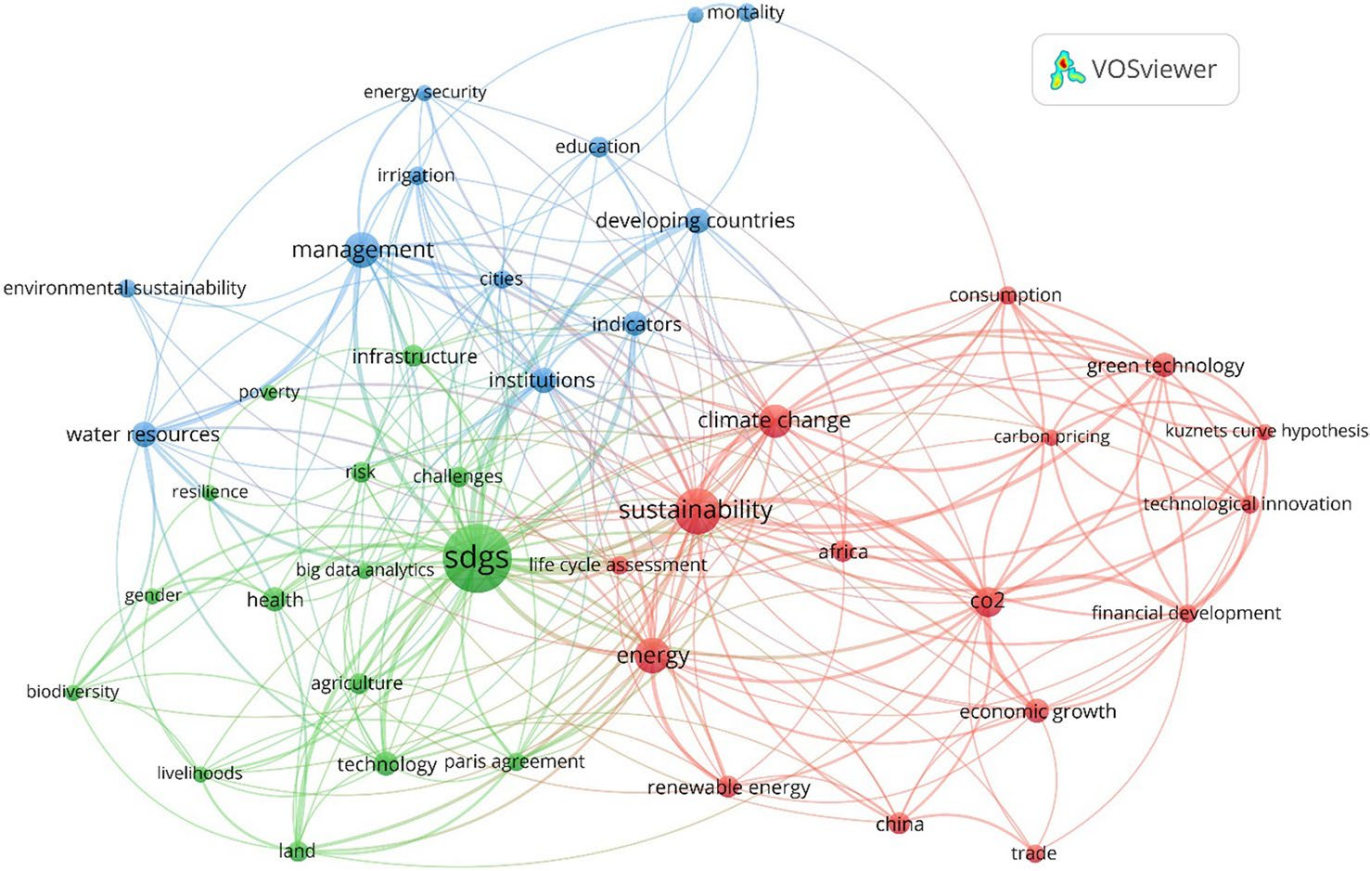
The output of the term co-occurrence analysis

Firstly, a bibliometric analysis was performed using the following syntax: TS = (((“SDG*” OR “sustainable development goal*”) NEAR/20 (“implement*” OR “operationali*” OR “achiev*”)) NEAR/20 (“resource*” OR “investment*” OR “technolog*” OR “infrastructure*”)). To obtain an overall understanding of the structure and thematic focus of research related to requirements in investment, new technology, and infrastructure to achieve the SDGs, we relied on the text mining abilities of VOSviewer, a widely used software tool for bibliometric analysis [61]. Among different outputs, the software allows for the understanding of key research focus areas by analysing bibliometric details of articles indexed in scientific databases. Given its broad coverage of quality research related to the study topic, the Web of Science was the scientific database used to retrieve relevant articles for this study purpose. To search for relevant articles, a broad-based search string was developed that includes terms related to SDGs, investments, technologies, and infrastructures. The initial search was done on March 19, 2021, and returned 407 articles. After screening the titles and abstracts of these articles, 154 were selected for final analysis using the VOSviewer. Major thematic areas were identified using the term co-occurrence analysis of the software. The output is shown in Fig. 3, where the node size is proportional to the occurrence frequency, and the link width is proportional to the strength of the connection. Terms that are closely related to each other form thematic clusters.

Secondly, it was necessary to assess the global progress of SDG implementation and the impact of the COVID-19 pandemic on it. This snapshot was done through “The Sustainable Development Goals Progress Chart 2021” [17], which covers the progress of selected targets under the 17 SDGs of the 2030 Agenda for Sustainable Development by the end of 2020. This document allows us to see how far we have come in realising global commitment and which areas require more attention. The charts presented are useful since they provide two kinds of information: (i) the trend assessment and (ii) the level of development assessment based on the latest data available, choosing the year 2015 as the baseline. After this assessment, the identification of SDGs levels of priority intervention was performed, through a scale of three points: high, moderate, and low. A high priority level was attributed to those SDGs that have an assessment of deterioration in any of their targets; a moderate priority level was attributed to those SDGs that have an assessment of moderate distance to the target, which highlights that some progress has been identified; and a low priority level was attributed to those SDGs that have an assessment close to the target, showing that they are accelerating target implementation. The rationale behind this process is based on the main principle of the UN 2030 Agenda [1], which highlights the integrated nature and indivisibility of the SDGs, meaning that progress in all of them is needed and the imbalance of one part weakens the rest. The impact of the COVID-19 pandemic on the requirements of investments, new technologies, and infrastructures for the SDGs implementation was tracked by the UNCTAD SDG Investment Monitor [62] and analysed in the World Investment Report [63]. This overview of the pandemic impact was further substantiated by the further analysis of several related case studies.

Thirdly, an assessment of SDG requirements of investment, new technology, and infrastructure for their acceleration was proposed, based on the literature review and on the UN “SDG Accelerator and Bottleneck Assessment tool” [64], developed to support nations worldwide to identify ‘accelerators’ that can trigger the SDGs implementation positively and have a multiplier effect across their targets. Through this, three levels of requirements were created regarding investment, new technology, and infrastructure, resulting in a matrix with these three parameters crossing, with different sizes for the importance of the above items for each SDG.

Fourthly, the identification of the main challenges (barriers/drivers) to the SDGs acceleration was performed to help the development of policy and/or programme areas—which will accelerate progress across the SDGs and the corresponding drivers that enable their progress. The final step to identifying how the SDGs that are part of each group are interconnected, i.e., its synergies, is to assess how the requirements of one of them can help to accelerate the remaining ones.

Fifthly, the last step of this methodological process was the systematic analysis of 11 individual case studies to triangulate the holistic analysis (Additional file 1: Table S1). Based on Yin [65], the cases were classified as type I because each one was analysed as a whole unit to show how the challenges of SDGs implementation have been overcome. This process allowed the authors to contrast the interpretation of the results and enrich the explanation of this complex study. Thus, the cases were determined based on four criteria that looked for the triangulation of the analysis, which are the following:

- Ensures an adequate representation of the SDGs based on their interconnected nature; therefore, three cases were selected about the food chain, which directly and indirectly discussed SDGs 1, 2, 3, 4, 5, 6, 7, 8, 9, 10, 11, 12, 13, 15, 16 and 17.
- Guarantees the analysis of the structural axis of the sustainable development model. Consequently, three cases about energy were selected; these showed the integrative nature of SDGs 1, 3, 7, 13, 14, 15.
- Secures the incorporation of the territoriality approach in the implementation of the SDGs. Four cases were selected to outline the application of SDGs 3, 6, 11, and 17 in Latin America, since it represents one of the most unequal regions with large technological, investment, and infrastructure gaps.
- Incorporates the discussion about a system of metrics to follow up on the SDGs in the framework of institutional heterogeneity of the countries. Thereby, the last case was selected.

## Results

### Bibliometric analysis and assessment of the global progress towards SDGs achievement

The results from the analysis of the term co-occurrence show that research on the investments, new technologies, and infrastructures that is needed for the achievement of the SDGs has focused on issues related to various SDGs (Fig. 3). For instance, there are frequently used terms related to climate change and emissions (SDG13); health and mortality (SDG3); institutions and management (SDG16); water resources (SDG6); agriculture (SDG2); energy (SDG7); poverty (SDG1); cities (SDG11); land and biodiversity (SDG15); education (SDG4); innovation (SDG9); consumption (SDG12); economic growth and circular economy (SDG8); and gender (SDG5). This signifies the importance of investments, technologies, and infrastructures as pivotal elements for achieving the SDGs. There has been more emphasis on terms related to climate change adaptation and mitigation, with related terms mainly being concentrated in the red and green clusters. This could be interpreted as increasing technological innovation and infrastructure development as tools for solving the societal challenges caused by climate change. However, the inclusion of terms related to the remaining SDGs shows that other socioeconomic aspects should also be considered. Technology is believed to play a critical role in this regard. Therefore, technological innovation should be aligned with the principles of sustainable development to contribute to meeting the needs of all societal groups and ensure development within planetary boundaries [66]. While technology is a major driving force of economic development, it has traditionally been dominated by a few giant companies that have prioritised consumption-oriented growth over sustainable development [66]. Out of all the different technologies discussed in relation to the SDGs, there has been a specific focus on digital technologies enabled by Information and Communication Technologies (ICTs). Such technologies are believed to promote economic development and improve national and local prosperity through optimising operations and improving service accessibility [67].

Table 1 aims to show the world’s progress on SDGs implementation.

**Table 1:**
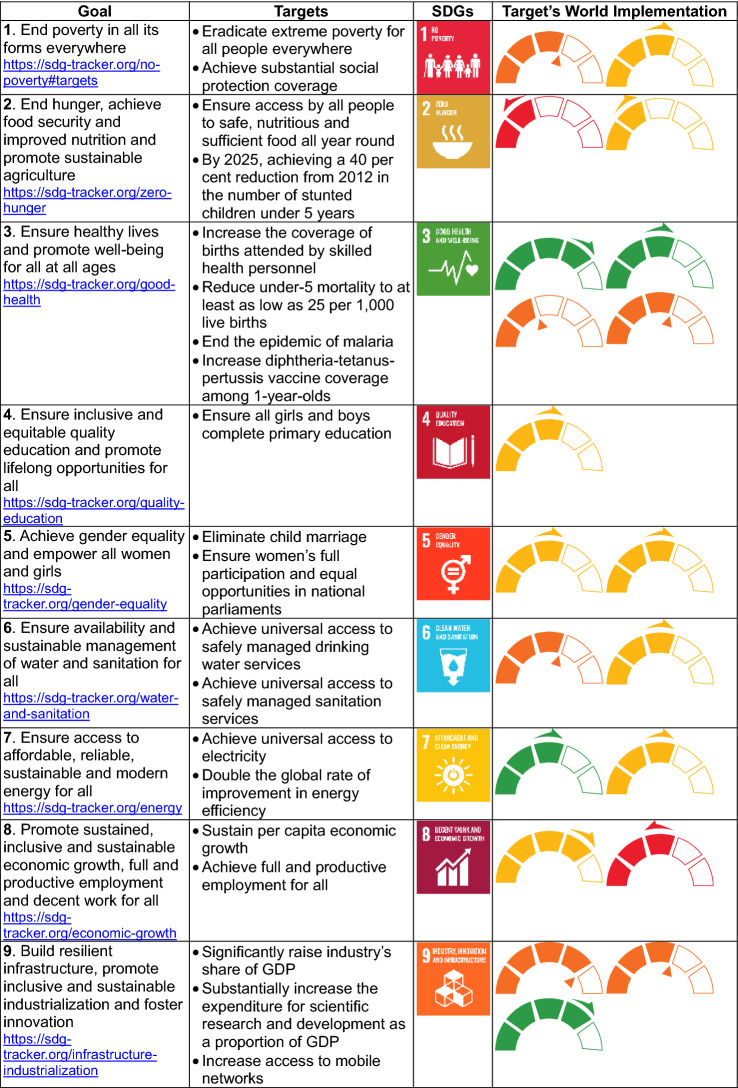

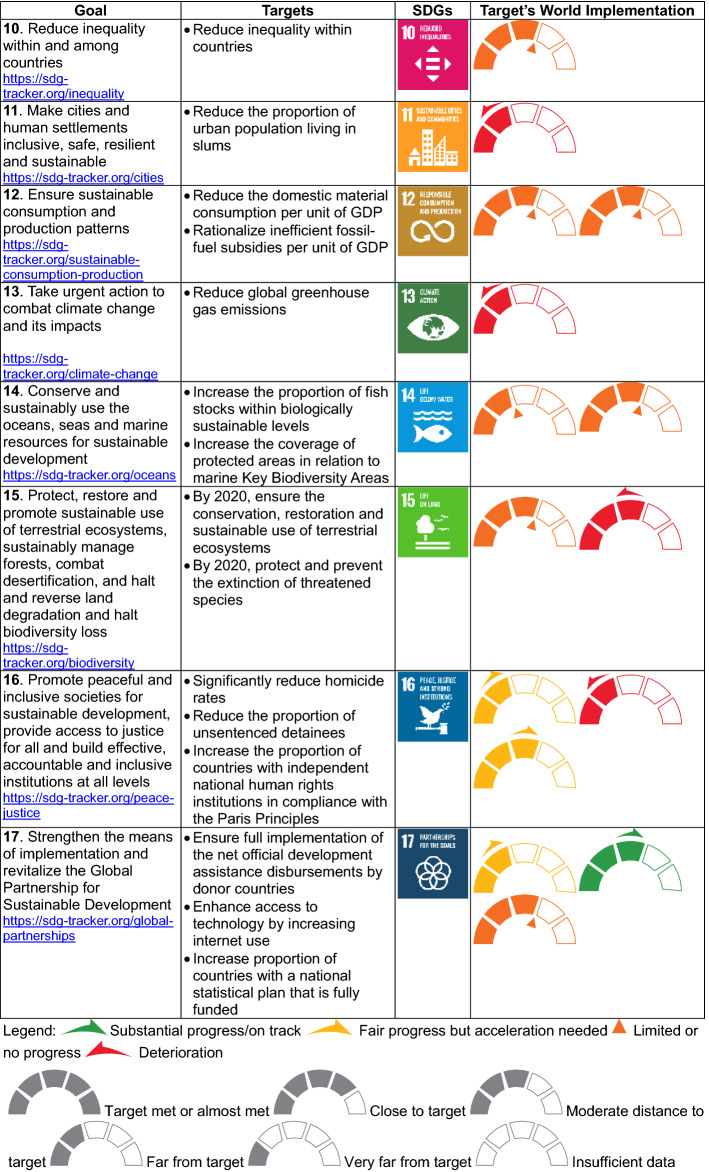
United Nations Sustainable Development Goals and world progress on them by 2021. Data obtained from the United Nations [17]

Data in Table 1 show that despite the relevant efforts worldwide toward the implementation of the SDGs, the pace of progress has been insufficient and not fully realised. Thus, a substantial acceleration is needed, especially for the five SDGs that have presented a deterioration since 2015: SDG2, SDG11, SDG13, SDG15, and SDG16. On the other hand, SDG3, SDG7, SDG9, SDG14, and SDG17 have shown substantial progress and are on track to be fully implemented. These results are quite interesting when cross-checked with UN reports [68], which state that SDG17 on global partnerships has received the most attention, being recognised by nations as central to Agenda 2030. Also, SDG13 on climate change has received the second most amount of attention according to the same source. However, our data show a deterioration, which may represent that this widespread recognition needs to be translated into action; something that is far from being fully realised. SDG10 has received the least amount of attention by nations worldwide and represents a structural challenge for the 2030 Agenda implementation since it is assumed to add a key impediment—a finding that is by itself highly concerning.

To further explore the SDGs that need acceleration, Table 2 presents the goals organised by priority level according to the level of requirements needed for investments, new technologies, and infrastructures.

**Table 2:**
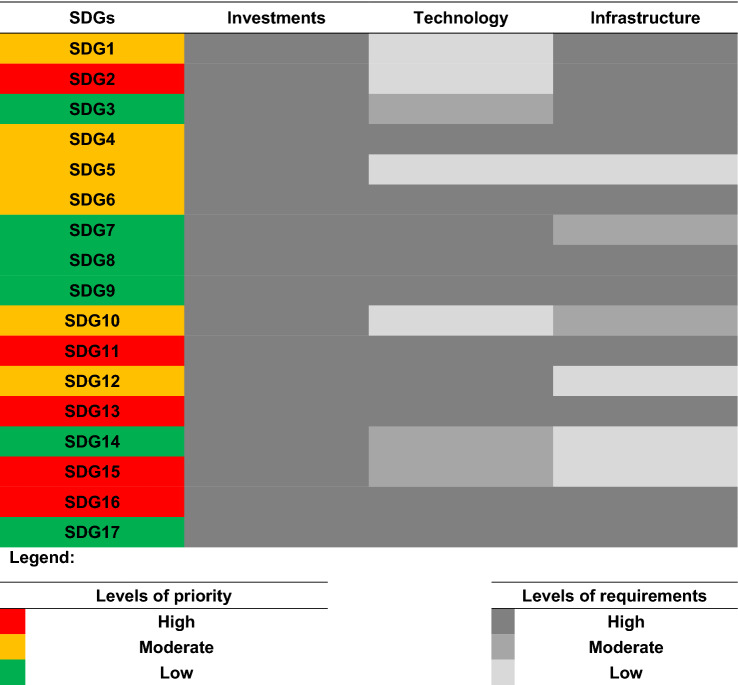
Matrix table regarding SDGs intervention priority level and requirements on investments, new technology and infrastructure

It can be stated that five SDGs were identified for high-priority intervention: SDG2, SDG11, SDG13, SDG15, and SDG16. SDG1 and SDG2 are universally recognised as the highest priorities at the global and regional levels [69]. However, despite some visible progress on SDG1, in relation to SDG2, the efforts are insufficient. Hunger is strongly linked with poverty, so synergies need to be created to combine efforts [53, 54]. Also, SDG13 and SDG15 are closely related [69], so it is not surprising that both presented a deterioration. In fact, the UN Secretary-General presented some priorities for 2021 [70] that are somewhat aligned with those identified in this study, namely, to make peace with Nature, tackle poverty and inequality and reverse the assault on human rights. All SDGs, independently of their priority level, depend on high investment for their implementation, but current evidence states that global investment is falling short of the target to close the gap of $2.5 trillion of annual financing, especially for developing countries [71].

Since infrastructure is a complex time-consuming task, it needs long periods of analysis in order to translate which actions were in fact able to allow for effective SDG achievement. Literature shows that actions are easily politicised [72]. Because the cost involved in infrastructures is mainly covered by the community, the engagement of citizens is crucial in response to societal problems, such as the ones necessary to be addressed in Table 1.

Table 2 clearly shows that the level of requirements towards investments to be made in SDG achievement is high in any case, at the global level. This is closely connected with the infrastructure requirements to be fulfilled, as discussed below. In fact, it can be stated that investment is at the core of infrastructure and technology development, as shown in Table 2, so without proper investment a deterioration of SDGs can be a negative consequence.

It is widely acknowledged that infrastructures are crucial to achieving significant development outcomes, i.e., poverty (SDG1), health (SDG3), education (SDG4), economic (SDG8) and environmental (SDG 12, 13, 14, 15) targets [73] through collaborative partnerships (SDG17) [74]. This can be of utmost importance, since many of these SDGs were identified as of high priority (Table 2). They profoundly influence progress [75]. Energy transport and waste, as examples, are part of essential infrastructure systems that support the economy, livelihoods and a sustainable planet. Accordingly, and in order to avoid strategic selection, the success of potential investments and policies must be guided by the relevant stakeholders, necessarily addressing the private sector and allowing for continuous adaptive assessment and progress through continuous change.

Vinuesa et al. [19] have produced a summary of the positive and negative impacts of artificial intelligence, documenting the potential of technology to act as an enabler or inhibitor of each SDG. The results show that environment (93%), society (82%), and economy (70%) are strongly and positively affected by artificial intelligence, whereas it acts as an inhibitor in society (38%), economy (33%) and the environment (30%). The enablement is mainly translated through technological improvement [76].

### Case studies

Complementing the bibliometric analysis, 11 case studies were outlined (provided as Additional file 2: Table S2) to show how these key challenges impact, either directly or indirectly, the pursuit of the SDGs. As the case studies illustrate, there is a tension between achieving the SDGs on the one hand, while meeting the climate targets set by the Paris Agreement, on the other. Substantial transformations are needed in technologies and infrastructured, which require huge investments in all areas. As Sachs et al. [3] suggest, transformations that can serve as the building blocks of SDG achievement can be grouped into six areas: (1) education, gender and inequality; (2) health, well-being and demography; (3) energy decarbonisation and sustainable industry; (4) sustainable food, land, water and oceans; (5) sustainable cities and communities; and (6) digital revolution for sustainable development. Table 3 illustrates these types of infrastructure and some of the investments required for the implementation of the SDGs.

**Table 3:** Key challenges in the implementation of the SDG

Case studies	Infrastructure	Investment	New technologies	Main impacts
1	Technological, regulatory, social, environmental and institutional	Initially very high as new technologies and grid infrastructure need to be developed	√	Socioeconomic, regulatory, policy, and environmental
2	Technological institutional, and regulatory	Initially low as research needs to establish metrics	√	Socioeconomic, policy, and environmental
3	Visual/landscape, environmental, socioeconomic, and procedural	Initially medium–low because its implementation by issues is gradual	√	Socioeconomic, regulatory, policy, and environmental
4	Technological, social, institutional and political	Initially medium because developing metrics is gradual	√	Socioeconomic, and policy
5	Technological, social, institutional and political	Initially low because its hybridisation with traditional systems	√	Social and economic
6	Technological, social, environmental, institutional and political	Initially medium–low because its implementation by issues is gradual	√	Social, economic and environmental
7	Technological, social and environmental	Initially medium–high because the regions need public resources due to social disadvantage	√	Social, economic and environmental
8	Social and political	Low because it does not refer to the cost of each project	√	Social (focused in territorial and human)
9	Social, institutional, political	Initially medium–low because its implementation is in line with local legal frameworks		Social and political
10	Social, environmental	Low, since the most important tool is raising awareness among food consumers		Social and economic
11	Technological, environmental and institutional	Initially very high as new technologies need to be supported by expensive research	√	Technological and economic

The complex networks of social, institutional, financial and political interactions were one common dynamic characteristic of the case studies. These coincide in their contribution to increasing the resilience of the populations and ecosystems involved, and to impacting in social, environmental, economic, political and regulatory ways. Based on this analysis, the greatest challenge refers to the infrastructure and investment. In the field of infrastructure, those that are needed are related to the technological, social, institutional and political context. Regarding the investments needed, the majority range from medium to high, highlighting that an effort to unlock the potential of technologies to accelerate SDGs implementation from companies, governments, industry, academia, and civil society is indeed costly, but also crucial. It is important to change the paradigm that is anchored in fragmented achievements to build upon a global investment of money, time, and expertise into the implementation of the UN 2030 Agenda.

From Table 3, it is clear that the main impacts that will derive from the investments in new technologies to support the acceleration of SDGs are mainly of socioeconomic, political, and environmental nature. This means that through the connection of citizens worldwide, new technologies are able to monitor and track the environmental and social impacts.

A further lesson from the case studies is that a “one approach may fit all” is not feasible when it comes to the implementation of the SDGs. Each goal is characterised by specific features, and addressing them requires distinct sets of resources, whose mobilisation is essential if they are to yield the expected benefits. There is at present a discrepancy between the value of investment needed and the available infrastructure needed in some areas. In order to address this problem, a balanced approach is necessary: in some contexts, investments in infrastructure are needed as a precursor to other types of investment.

## Discussion

Table 4 aims to identify the main challenges to implementing SDGs as well as the need for investments, new technologies, and infrastructures to accelerate it.

**Table 4:**
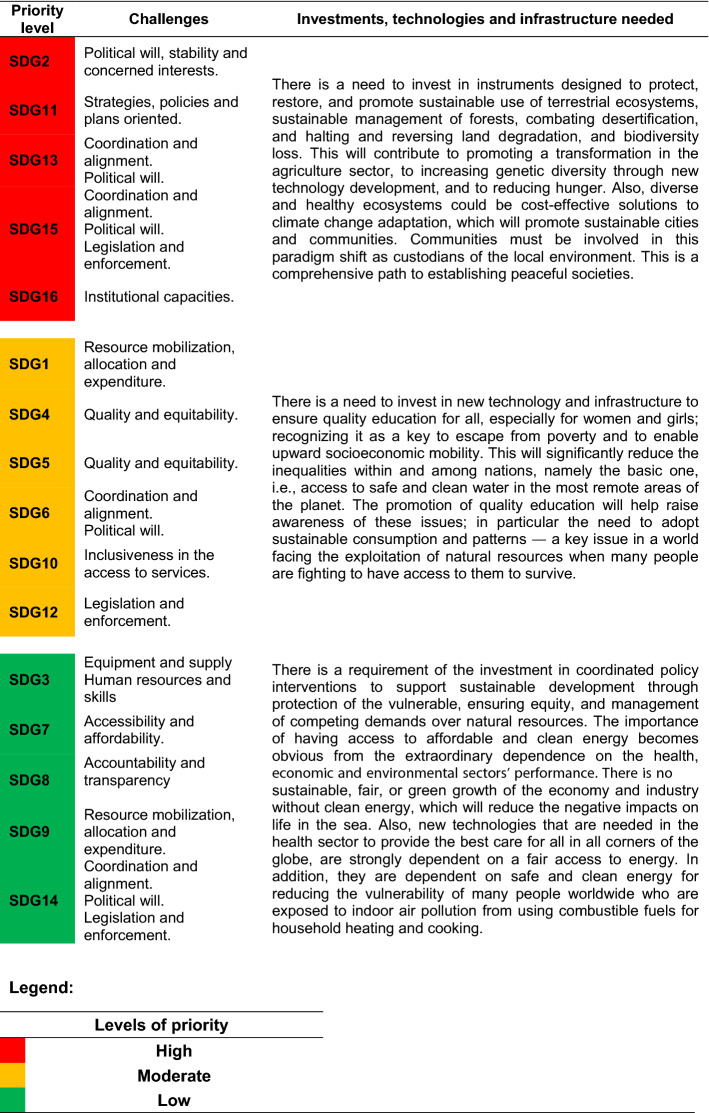
Identification of the main challenges and requirements on investments, new technology and infrastructure by SDGs priority intervention level group

Regarding Table 4, SDGs that need a high-priority intervention (red colour) are those that most depend on political will and coordination and alignment. Poverty and hunger are mainly related to food security [77], which can be alleviated through a transformation in the agriculture sector [78]. The use of advanced technologies and practices is critical to intensifying food production sustainability, namely in disadvantaged communities that are more vulnerable to poverty, hunger, and food insecurity [79–82]. This is also a key issue regarding climate change adaptation, which consequently can result in significant gains in promoting sustainable cities and communities [83, 84].

The high priority level indicated in Table 3 mostly addresses the more vulnerable countries, which are the focus of international assistance [85]. While it can be stated that the SDGs agenda may suffer from political influence, the needs are identified in relevant international literature. How to get there in each case is another question. If the focus is many times centred on the social dimension (SDGs 1 to 7, 11, and 16), the economy (SDGs 8 to 10, 12, and 17), and environmental dimensions (SDGs 13 to 15) are seen to be major issues worldwide. Critical choices must be made to specifically address the multiple dimensions of sustainable development. An analysis of achievements clearly shows that everything goes well with rich developed countries and almost everything goes wrong with the poorest. The problem thus lies in the poorer countries, affecting the SDGs scores at a global level [86].

Investment is crucial to achieving the SDGs in regions worldwide. In developing countries, where achievements towards the SDGs are known to be under 10% in specific regions for a significant number of SDGs (1 to 5, 7, 9, and 14), foreign investment is seen as an external financial source in the private sector, beyond the public investment that influences SDG scores [87]. The consequences are especially relevant at the level of basic infrastructure, clean water, sanitation (SDG6), and renewable energy (SDGs 7).

According to Vinuesa et al. [19], SDG1 (poverty), SDG4 (quality education), SDG6 (clean water and sanitation), SDG7 (affordable and clean energy), and SDG11 (sustainable cities) illustrate examples where artificial intelligence can act as an enabler of sustainable development. Looking at Table 3, we can see that the indicated SDGs mainly address the moderate and low-priority groups. Interestingly, SDG11 on sustainable cities is included in the high-priority group, illustrating the importance of technology in contributing to necessary sustainable development. Other SDGs, such as SDG 12 (climate action), will clearly benefit from interconnected technologies aimed at achieving environmental transformative changes through low-carbon cities, for example. The negative impacts of technology can be perceived in the societal sphere when technology is implemented in countries without access to huge computing centres and with a consequent high footprint, compromising SDGs relevant to the environmental sphere and triggering inequalities inhibiting SDGs 1, 4, and 5, as an example.

Developing countries, where technology is highly insufficient, both in terms of investments to be attained as in terms of human capital, are a clear example of inequalities. If the situation requires additional qualifications to work with technology, it will on the other hand increase associated inequalities. How to achieve balance in the successful implementation of investment, infrastructure and technology is an extremely difficult task. Technology is strongly linked to the needs of a particular regional context, and there is no global solution available. It is unevenly distributed and available everywhere, inhibiting the access to SDG2, for example, or SDG5, regarding minorities. On the other hand, technology can be used to increase productivity, positively contributing to economic growth, but can also exacerbate inequalities, which is, in fact, the major problem worldwide, significantly impacting SDGs 8, 9, and 10. Infrastructures is the dorsal spine of society, including energy, water, waste, transport, or communications.

Investments made are mostly focused on economic development but they have consequences on the SDGs achievement at the global level [88]. Incorrectly planned provisions of infrastructure are able to negatively affect the desired outcomes, namely in terms of human health—through pollution (SDGs 14 and 15), for example. While studies focus on the relationship between infrastructure and the economy, policies to stimulate the change in infrastructure use, supported by investments, will contribute to the sustainable supply.

Regarding SDGs that need moderate intervention, resource mobilisation and allocation, as well as quality and equitability, are the main challenges to their implementation. The interconnection between them—their synergies—should comprise the premise that education is a pivotal dimension that can help to reduce poverty and empower women and girls. Barbier and Burgess [89] have demonstrated that the SDGs, in particular SDG1, can be boosted with positive gains when improvements on SDG6 were made. Also, the acceleration of SDG1 and SDG6 implementation will contribute to reducing inequalities within and among countries (SDG11). Relatedly, Pradhan et al. [90] argued that SDG1 holds a synergetic relationship with most of the other SDGs, and that should be considered as a potential accelerator of the UN 2030 Agenda implementation.

The low-priority group requires coordinated policy interventions to protect the vulnerable ones, by ensuring equity. This is also applicable to the management of competing demands over natural resources to support sustainable development, especially in what relates to SDG3, SDG7, and SDG14 [91]. The investments and infrastructures needed to improve the access to affordable and clean energy become obvious from the extraordinary dependence on the health, economic and environmental sectors’ performance. As can be seen, energy, which is omnipresent in our lives, plays a decisive role in many SDGs. Thus, it is a positive outcome to see that SDG7 is part of a low-priority group intervention.

The assessment of investments, technologies, and infrastructures required for the implementation of SDGs needs to pay particular attention to the unprecedented impact of the COVID-19 pandemic. Despite the progress that was made since the SDGs were adopted in 2015, the impacts of the COVID-19 pandemic and the measures of controlling the pandemic on SDG implementation were found to be devastating [63]. According to the UNCTAD *SDG Investment Monitor* [62], SDGs investment activities declined sharply across all SDGs sectors, except for renewable energy, which continued to grow in new SDGs projects, however, only at one-third of the pre-COVID level. The international project investment in infrastructure and infrastructure industries (including utilities and telecom) fell by 62%. Investments in food and agriculture, water and sanitation, health and education, were reduced by one to two-thirds from 2019. More importantly, developing and transition economies suffered much larger reductions in SDG-related investment than developed countries. SDG-related investment declined by 51% in Africa, 44% in Latin America and the Caribbean, 33% in Asia, and 27% in transition economies. Overall, the progress made in promoting SDG investment since 2015 when the SDGs were adopted was found to be more than wiped out. By the end of 2020, the health crisis caused the investment in SDG sectors in developing and transition economies to drop 27% below the level from 5 years ago and the international project finances to drop by 12% [62].

The COVID-19 pandemic situation in which we are currently still living is a stark example of the uncertainty that the world could have never imagined being possible. Its gravity and persistence will force the humanity and research community to produce thousands of studies worldwide, focusing not only on the consequences it has on the wide-reaching SDGs achievement, but also considering it as a new uncertainty factor for future SDGs implementation and the necessary increased requirements of SDG-relevant proactive, preventive and adaptive strategies and resources.

This study examined the impacts of the COVID-19 pandemic on the implementation of SDGs thorough a review of the existing related research. The results of the examination indicate that not only the COVID-19 pandemic caused enormous mortality, health issues, and socioeconomic disruptions, but the measures taken to fight against the COVID-19 pandemic, such as lockdowns and travel bans, also had unprecedented impacts on the SDGs. While SDGs implementation faces challenges in all nations, the COVID-19 pandemic hit the poorest countries the most. Energy transformation slowed down in Europe, China, and India, but the livelihood of the poorest residents in the least developed countries, such as sub-Saharan Africa and Nepal, has dropped significantly below the level before the COVID-19 pandemic. At the same time, the recovery from the current pandemic and the prevention of future crises will also be translated into increased requirements for investments, new technologies, and infrastructures. The results of the investigation are outlined in the supplementary material (Table A2).

## Conclusions

This study addresses the question as to which investments, technologies, and infrastructures are needed to assist in the implementation of the SDGs and examines the extent to which they are available, also considering the current limitations caused by the still ongoing COVID-19 pandemic. It pursued its aims by adopting a synergy of different methods, which included a bibliometric analysis, an assessment of the global progress of SDG implementation, requirements and challenges, and a set of eleven case studies to triangulate the holistic analysis.

Overall, the findings suggest that the scope and width of resources limitation are currently undermining the implementation of the SDGs. Apart from that, is the fact that the pace of progress has been insufficient and the potential of the goals in pursuing sustainability and improving life quality is not being fully realised. This trend suggests that a substantial acceleration of the efforts is needed, especially for the five SDGs whose progress since 2015 has not been optimal, in particular SDG2, SDG11, SDG13, SDG15, and SDG16. It is also noticeable that SDG3, SDG7, SDG9, SDG14, and SDG17 are showing signs of progress. In both antagonist cases, improvement in respect of investments, coupled with the provision of technologies support and infrastructure could contribute to support future progress. In addition, the case studies showed that different industries have different effects on achieving the goals, i.e., the food sector directly or indirectly correlates with 15 SDGs, as opposed to the energy sector correlating with 6 SDGs. Finally, the priority level assessment in terms of achieving the SDGs point to the following SDGs: 2, 11, 13, 15, and 16. As perfectly evident until today, the COVID-19 pandemic has had severe impacts on the SDGs implementation process, in the ways outlined through this study.

This study has some limitations. One of them is the fact that the analysis focused on the availability of investments, technologies, and infrastructures and their importance does not equally apply to all SDGs, since some of them do not depend equally on these factors. The second limitation is that the study reports on the available international literature, and does not specifically entail individual country experiences. However, despite these limitations, this study provides a contribution to the literature in respect of the fact that is presents evidences of the negative impacts of resources deprivation, and systematically points out the necessary actions to mitigate them.

Relying on an extensive literature collection, the main implications of this study are that it fills in a knowledge gap in respect of the current need for and availability of investments, new technologies, and infrastructures to allow countries to pursue the 17 SDGs. The findings resulting from the substantial analysis carried out suggest that this availability is rather limited in some contexts, shedding light on the various financial, technological and infrastructural needs to achieve the SDGs.

Since the global progress in the process of implementation of the SDGs depends directly and indirectly on addressing the resource gaps, it is suggested that these issues will be further investigated, so that the present imbalances in the three dimensions of sustainable development, i.e., the economic, social and environmental, be entirely addressed.

## Supplementary Information

**Additional file 1: Table S1.** Systematic analysis of 11 individual case studies to triangulate the holistic analysis

**Additional file 2: Table S2.** Impacts of the COVID-19 pandemic on the implementation of SDGs.

## Data Availability

Not applicable.
