# Supplementary material for: An assessment of requirements in investments, new technologies, and infrastructures to achieve the SDGs
Source: Environ Sci Eur. 2022 Jul 1;34(1):58. doi: 10.1186/s12302-022-00629-9 (PMC10127159; doi:10.1186/s12302-022-00629-9)
Supplement: Supplementary file 1 — Additional file 1: Table S1. Systematic analysis of 11 individual case studies to triangulate the holistic analysis [file 12302_2022_629_MOESM1_ESM.docx]

Table S1. Systematic analysis of 11 individual case studies to triangulate the holistic analysis

| *Case 1. Infrastructural challenges (associated with legislation and other requirements) in implementing SDGs in the food chain.* |
| --- |
| Since the adoption of the UN SDGs in 2015, policy makers and governments adopt legislative requirements to achieve sustainable development (SDG 17) at different paces. One of the initiatives is the upcoming Food Systems Summit that will be convened by UN Secretary-General António Guterres, where it is expected that various actions related to all 17 SDGs will be launched and associated with healthier and more sustainable food systems [66]. Regarding food systems, infrastructure in terms of legislation and standards is necessary for all types of conformity assessments, such as testing, inspection, validation, verification, certification, and accreditation [67] related to UN SDGs. These assessments should ensure sustainable connections between people and the planet during growth (SDGs 1, 2, 3, 13), processing (SDGs 2, 6, 7, 12, 13), transportation and storage (SDGs 7, 11, 12, 13), trade (SDGs 2, 3, 12) and consumption of food (SDGs 2, 3, 12) [68].The International Organization for Standardization also promotes their commitment to sustainable development with the connection of published standards and UN SDGs. The technical committee ISO/TC 34 'Food products', which is in charge of developing and publishing food related standards [69], declares contributions to the following SDGs: 1, 2, 3, 5, 8, 10, 11, 12, 13, 15 and 16. Another approach to expressing commitment towards sustainability is by reporting corporate social responsibility practices [70]. Currently, such examples may be seen only with multinational food companies that communicate their connection with the UN SDGs in their CRS reports, such as Nestle [71], Ferrero [72] or Danone [73]. |
| *Case 2. Role of food consumers in achieving SDGs as the last link in the food chain continuum pipeline* |
| Sustainable dietary patterns correlate with zero hunger (SDG 2), contribute to health (SDG 3) and climate change (SDG 13) and achieve sustainable consumption (SDG 12). Main dietary patterns are confronted between plant-based and animal-origin based diets as two extremes [74], with a variety of eating regimes in between (raw foodism, fruitarianism, vegetarianism, veganism and/or flexitarianism). In addition, there are also 'territorial' diets, with some pronounced as 'the healthiest’, like the Mediterranean cuisine [75], or religious schemes (fasting in Christianity, Halal in Islamic communities or Kosher linked with Judaism) with their requirements regarding food production [76]. However, pressure on changing eating habits towards sustainable diets is associated with environmental footprints and is more explored in scientific models than observed in everyday life [77]. Besides food consumption, food waste is also associated with this part of the food supply chain. The life-cycle approach of food systems usually points out food waste as an element that occurs in all food systems where households are recognised as significant contributors [78, 79]. This additionally stresses the need for achieving SDGs 12 and 13, as food waste has a pronounced carbon footprint. Therefore, it is necessary to analyse social and cultural habits from food purchasing to food preparation to waste disposal and to raise awareness among household members about food waste, mainly because discarding food waste is correlated with expiration dates, plate leftovers and spoiled food [80]. Therefore, achieving zero hunger (SDG 2, 3) and decreasing food waste (SDG 12, 13) should be understood as two different sides to a coin, and solving these issues will promote food sustainability [76]. |
| *Case 3. Technological challenges of implementing new non-thermal food processing technologies to achieve the SDGs* |
| Non-thermal food processing technologies are currently being studied in various domains of food application, such as food product stabilisation, extraction, or alternative preservation, and display several benefits: shortened treatment time; prevention of negative heat effects on nutritional characteristics; and satisfactory levels of food safety with maintained food quality attributes [81]. Within the food industry, different technologies have been used depending on their mode of action and energy source such as technologies based on mechanical action (hydrodynamic effects, ultrasound and irradiation); electro-magnetic fields (pulsed fields/ultraviolet light, cold plasma, oscillating magnetic fields) and pressure (high-pressure processing or supercritical fluid drying). However, the main challenge in applying these technologies within the food supply chain is their technology readiness level, as most of them are still at lab-scale with levels between 3-5 based on the EU scale [82]. Therefore, it is necessary to upscale the lab-scale food processing technology to an operating level (for benchmarking with conventional food processing technologies) and further upscale it to analyse the entire food supply chain. This is one of the reasons for the limited number of research papers with measurable sustainable parameters [83–85]. However, current knowledge confirms that these new technologies minimally process food and have a lower total sustainability impact, such as on water and energy consumption (associated with SDGs 6 and 7), achieving an overall lower carbon footprint (SDG 13) and striving toward sustainable production (SDG 12) [86]. Based on their beneficial nutritional characteristics (SDG 3), such products have the potential of combatting malnutrition and food insecurity (SDG 2) as Zero Hunger is identified as the key SDG associated with the food supply chain [68]. In order to achieve these goals, it is important to educate a new generation of sustainable food experts (SDG 4). |
| *Case 4: Offshore wind development needs technology investments, new policy frameworks, and improved institutional capacities* |
| Renewable energy (RE) has been recognised as a key factor in reducing global GHG emissions and climate change mitigation (SDG13). RE capacity continues to grow; more than 80% of all new electricity capacity added in 2020 was renewable, with solar and wind accounting for 91% of new renewables [87]. Offshore wind is a major contributor to renewable installations, especially in Europe. The European Union plans to have 300 GW of installed offshore wind by 2050—an ambitious target when compared to the existing capacity of 25GW. Such large-scale deployment of RE technologies requires multibillion dollar investments, expert knowledge, and skills development. In that respect, in 2020 alone the European offshore wind sector attracted over 26 billion euros ($31.7 billion) of investment—a record amount. Moreover, such RE developments require new policy frameworks and inter-agency coordination, especially when siting spans across borders. Recently, the European Union adopted its Offshore Renewable Energy Strategy, which envisions future offshore wind market developments in Central and Eastern Europe. The dynamics of this market development have been accelerating significantly. Poland’s first Offshore Wind Act was enacted on February 17, 2021, paving the way for the country to develop the largest market for offshore wind in the Baltic Sea. Romania is drafting its first Offshore Wind Law, offering support schemes for investors and tapping into the potential of the Black Sea, while Bulgaria is planning to add 16 GW of new offshore wind capacity by 2050 and to reform the country’s regulatory framework to accommodate the flow of investments. It is now more important than ever that investors recognise new opportunities to speed up the adoption of offshore wind projects [87–91]. |
| *Case 5: Besides investments in technology, RE deployment requires a research agenda that ensures that climate goals and SDGs are mutually reinforcing* |
| Scenarios that incorporate the SDGs in their calculations for keeping global average temperatures below 1.5^o^C above the preindustrial levels show the need for annual investments in renewable energy to reach $635 billion per year between 2025 and 2030. For comparison, the annual renewable energy investment for the period 2015-2020 was $308 billion per year (2019 equivalent dollars) [92]. This doubling of investment is needed for the production of new solar panels and wind turbines in order to increase energy access and affordability (SDG7) and to improve social and economic development (SDG1). Moreover, because of the interconnectedness of the SDGs, it is also needed for reducing renewable energy’s negative impact on the environment (SDGs 14 and 15) and on human health (SDG3). Research shows, however, that investments in renewable energies most often provide funding for just the technology’s research, development, demonstration, and deployment (RD3), but they do not provide funding for examining the environmental impacts of those technologies. Some issues related to the deployment of RE technologies, which are difficult to quantify, are the negative impacts on wildlife and on human health (noise caused by wind turbines and glare by solar panels), biodiversity loss, soil contamination, mountain top removal, or siting issues related to environmental justice. Such issues are common to the traditionally used fossil fuels, but this does not mean they should not be accounted for in the deployment of renewables. A good starting point is to set up a research agenda for technology research, development, demonstration, and deployment (RD3). Such an agenda could be developed at workshops, such as the one organised and described by Hernandez et al. [93]. During a two-day workshop in 2019, 58 expert stakeholders gathered to construct a comprehensive roadmap for solar and wind energy so as to anticipate and improve impacts of the transition to a low carbon future in a manner that ensures that the climate goals and SDGs are mutually reinforcing. [92, 93]. |
| *Case 6: Public acceptance of renewable energy technologies is key for achieving the SDGs* |
| Public acceptance of RE technologies has been identified as a key to the successful implementation of renewable energy projects and decarbonisation (SDGs 7 and 13). Unfortunately, there is a widespread, persistent, and not-necessarily conscious view (among some developers, researchers, and policy makers) that local resistance is irrational, selfish, or ignorant. Research shows that most often the community concerns are valid and need to be taken seriously. A framework has been proposed for understanding and dealing with community concerns [94]. Called VESPA, the framework stands for the Visual/landscape, Environmental, Socioeconomic, and Procedural Aspects that are the positive and negative factors impacting acceptance among the members of the local population. When RE developers intentionally consult with the local communities from the project design phase to its completion, when they are attentive to and address local concerns, when they do not overpromise and are willing to give back to the community, they are able to garner trust and achieve greater public acceptance and support. This leads to faster siting and deployment, savings for the developers and investors, and achievement of the fundamental transformations needed to meet the SDGs, with all stakeholders involved at each stage of the transformation process in order to design pathways and achieve wider buy-in from all participants at the government, business, and civil-society levels. [94–96]. |
| *Case 7. Ensure healthy lives and encourage welfare for all: Highly integrated environments and technologies 4.0 for digital transformation in the health sector* |
| The intersection of the cyber-physical systems with the health sector is called Health 4.0; this intends that the massive use of cloud computing (on the border), Internet of Things (IoT) and next-generation mobile communication networks are at the service of health and healthcare. Health 4.0 makes it possible to convert social challenges related to diabetes, antibiotic resistance, viral outbreaks, cancer, among other degenerative chronic diseases, into techno-economic opportunities to improve social welfare in Mexico. Mexico faces complex problems in the health sector; the high rate of overweight in the population and diabetes, among other health conditions, represent high public health risks that demand a disruptive renewal of the Mexican health system. This specific case showed that it is possible to strengthen the digital transformation of health and healthcare sectors in Guanajuato (Mexico), which despite the heterogeneous digital capabilities, it has a health system with a coverage of 95,8% in the supply of medicines, a continuous investment in health infrastructure, universality policies and free medical services, and a self-sustainable model with no financial debt. For instance, artificial intelligence is used to prevent breast cancer, or bioengineering to prevent the amputation of diabetic foot. The financial health of Guanajuato’s health system and the available infrastructure support the feasibility of the adoption of Health 4.0. However, significant social barriers, particularly the limited access to health services, the poor technology skills and the distrust from the most vulnerable segment of the population poses a major challenge to Health 4.0, possibly greater than the economic and technological restrictions [97–102]. |
| *Case 8. Transform the urban model towards more compact cities, and generate innovation and governance tools for the adaptation of human life based on new technologies* |
| The city is a strategic space for the achievement of the SDGs, and its organisation directly impacts on health, quality of life, and economy, among others. However, these manifestations strongly influence socially disadvantaged populations or countries with fewer response resources, which widen the gaps and accentuate social inequality. Among the technologies that mitigate the negative externalities of the city are distributed renewable energy, electro-mobility, and the circular economy or digital technologies that analyse large volumes of data to make decisions about the interactions between biophysical and socioeconomic variables through algorithms of artificial intelligence and deep learning. This specific case showed that cities such as Medellín (Colombia), Curitiba (Brazil) or Montevideo (Uruguay) execute participatory projects based on technology (for instance, Route N, WithCuritiba or AJE-Uruguay), in which multiple actors and sectors -productive, academic, institutional, financial and civil- interact to influence the transformation of their development model and focus its efforts to form inter-institutional and multi-sectoral groups. It is relevant to highlight that their universities have a dynamic interaction with the environment. Moreover, all collective actors generate interdisciplinary knowledge and social, scientific and technological actions in the face of humanity's vulnerability and lukewarm progress in the equitable achievement of the SDGs [103–106]. |
| *Case 9. Ensure the availability and sustainable management of water for all: agroecological practices in subsistence systems that have transformed rural regions* |
| Ecological technologies are rational artefacts that achieve a social relationship consistent with environmental impact; however, the dialogue of technical and local knowledge is strategic in maintaining cooperative and trustworthy environments with a technological memory that ensures its continuous and standardised use. The ecological history of LATAM shows that socio-institutional efforts have been boosted to respond to the social demand for water. Nevertheless, the results have not been as expected, not only because of the water stress in most of the region, but because the technology has not been socially adopted to facilitate the access and guarantee its rights. This specific case showed that it is possible to mitigate physical and economical scarcity through communitarian practices of management with a hydro-social approach in Cali (Colombia), Cuzco (Peru) and San Juan (Argentina) to manage the access or the extraction for irrigation. For instance, based on the mixture of technical knowledge, vernacular and ancestral practices of coexistence with nature, the ecotechnologies have encouraged that some committees manage from collective harvesters (Cali and San Juan) to a basin (Cuzco); this approach has had relevant results in these regions due to the share of resources for their survival and economic development, and it considers the social and power along with the legal and cultural aspects. A fundamental factor is the provision of public resources, which is necessary to incorporate ecotechnologies in regions that are not able to satisfy a basic line of welfare, and another is the local technological capability as the key factor to achieving the SDGs in these localities [107–111]. |
| *Case 10. Strengthen the collaboration and revitalise the interactions: communities, social innovation and local capabilities for sustainable development* |
| Sustainability exhibits strong challenges and contradictions that are anchored to the territories, from relational complexity to territorial capital. According to the literature review, the achievement of the SDGs has a high correlation with three main factors, which are the following: the territorial approach in the development processes; the local/community space approach to anchor the change processes, and the inter-territorial collaboration as an operational strategy. This specific case showed how the integration of a network of local actors (communities) from 17 countries in Latin America and the Caribbean (CODE-LAC network) allows them to share their endogenous experiences that are focused on achieving the SDGs through a strategy of experiential learning, which has as its main axes: inclusive governance to synchronise actions with structural change; social dimension of innovation as a structure in the processes of change; and centrality on reducing inequalities –cultural, gender - in social mobility, in access to resources, among other opportunities for equity. The initiative to trigger this network was born in the framework of the LEED Program (OECD), and since 2010, more than 300 Latin American local development agents have been getting involved to share knowledge, compare perspectives and identify best practices. This knowledge alliance has strengthened the actors to advance towards the achievement of the SDGs and to guide and reinforce the territorial development. Furthermore, this has boosted the dynamic capacities of the territory, which are decisive to achieving sustainable development (SDG) through the implementation of social actions and operative policies focused on taking advantage of, among others, social, cultural, scientific, economic, natural and institutional resources in Latin-American territories [112–114]. |
| *Case 11: Introducing a system of metrics for measuring global progress toward achieving both the SDGs and the climate targets set by the Paris Agreement* |
| Experts note the need to develop a comprehensive roadmap and coherent policies to ensure that both climate goals and SDGs can be achieved. In addition, since a huge portion of the investments come from companies, there is a need to measure their contributions to the achievement of the SDGs. Scientists have identified a gap in this area—no metrics exist that explicitly link company activity, largely from the sale of individual technologies (e.g., wind turbines, solar panels, and more fuel-efficient engines for vehicles) to an objective, scientific, data-driven assessment of how companies are contributing toward the SDGs. As it turns out, the way SDGs are incorporated in company reporting is complicated. Companies that use ESG (environmental, social, and governance) metrics as indicators of performance do not necessarily present all of their impacts. Typically, companies that focus on RE production count their contribution to SDG7; however, they could also count them towards clean cooking and wellbeing (SDG3), reducing gender inequality (SDG5), and CO2 reduction (SDG13). A proposed solution is the creation of a simple-to-use yet comprehensive system of metrics to measure global progress toward achieving both the SDGs and the climate targets set by the Paris Agreement. The incorporation of such metrics in models and frameworks is not an easy task and has many caveats—availability and standardisation of reported data, metrics that although tied directly to SDG indicators do not include the full life cycle of both positive and negative impacts or links to other SDG targets. However, as data become available and standardised, such models will be able to provide better temporal and geographic resolution, and become more useful not only for investors but also for institutions, cities, and governments [93, 115]. |
