# Supplementary material for: An assessment of requirements in investments, new technologies, and infrastructures to achieve the SDGs
Source: Environ Sci Eur. 2022 Jul 1;34(1):58. doi: 10.1186/s12302-022-00629-9 (PMC10127159; doi:10.1186/s12302-022-00629-9)
Supplement: Supplementary file 2 — Additional file 2: Table S2. Impacts of the COVID-19 pandemic on the implementation of SDGs. [file 12302_2022_629_MOESM2_ESM.docx]

Table S2. Impacts of the COVID-19 pandemic on the implementation of SDGs.

| **Case** | **Title of the case study** | **Short description** | **Implications** | **Studies** |
| --- | --- | --- | --- | --- |
| 1 | The implications of the Covid-19 pandemic for delivering the Sustainable Development Goals. | This study examined the likelihood of achieving the foundational SDGs in each of the four scenarios after the COVID-19 pandemic: a) global well-being prioritized; b) world trade recovers; c) poverty gaps widen; d) earth systems in danger. | Global responses to COVID-19 will impact on delivery of the 17 Sustainable Development Goals by 2030, creating large uncertainties, especially in terms of the competing priority between people's health well being and livelihood, just at the time efforts need to be accelerated. | Fenner, R., & Cernev, T. [1] |
| 2 | The role of flexibility in the light of the COVID-19 pandemic and beyond: Contributing to a sustainable and resilient energy future in Europe | Focusing on Europe, this study derives five urgent policy recommendations for Europe that address the impacts of COVID-19 on the economic and societal prerequisites for flexibility in energy systems. | Fighting against the COVID-19 pandemic, a well-functioning and resilient energy sector is vital for maintaining the operation of critical infrastructures, including, most importantly, the health sector, and timely economic recovery. | Heffron, R. J., Körner, M.-F., Schöpf, M., Wagner, J., & Weibelzahl, M. [2] |
| 3 | Nepal at the edge of sword with two edges: The COVID-19 pandemics and sustainable development goals | This study found that the COVID-19 pandemic has thwarted Nepal's targets to achieve the SDGs, and the populations below poverty could rise even more than the present national reported value of 18% in the post-pandemic era. | It urges to build international consensus to reset and rethink the course of sustainable development goals. | Joshi, T., Mainali, R. P., Marasini, S., Acharya, K. P., & Adhikari, S. [3] |
| 4 | Emerging and practical food innovations for achieving the Sustainable Development Goals (SDG) target 2.2 | This study focuses on the negative impact of the COVID-19 pandemic SDG 2.2 ending malnutrition as it currently exacerbates the triple burden of malnutrition, negatively affecting human health and the society. | The study indicates the urgent need to intensify applied food science and technology research towards addressing all forms of malnutrition and put the existing knowledge into practice to meet the SDGs at the deadline of 2030. | Mensi, A., & Udenigwe, C. C. [4] |
| 5 | Regional impact of COVID-19 on the production and food security of common bean smallholder farmers in Sub-Saharan Africa: Implication for SDG's | This study found COVID-19 to have caused significant challenges to the bean production across the sub-regions, including low access to seed, farm inputs, hired labor, and agricultural finance and to have reversed the gains made in the achievement of DSGs 1 and 2. | Sustainable and resilient food systems need to be built through strengthening and enabling public-private partnerships and direct investments from the government are necessary in supply systems and short food supply chains through digital access. | Nchanji, E. B., & Lutomia, C. K. [5] |
| 6 | Three pillars of sustainability in the wake of COVID-19: A systematic review and future research agenda for sustainable development | This study examined the current research on the COVID-19 impact on the SDGs and the impact itself and the research gaps and future research avenues for sustainable development post COVID-19. It found the need of alleviating the pandemic’s negative impacts on sustainable development and realizing the sustainability transition opportunities post COVID-19. | It calls for refining SDGs and targets and developing measurement framework, in-depth analyzing the COVID-19 long-term effects on social sustainability, and expanding quantitative research to harmonize the COVID-19-related sustainability research. | Ranjbari, M., Shams Esfandabadi, Z., Zanetti, M. C., Scagnelli, S. D., Siebers, P.-O., Aghbashlo, M., Peng, W., Quatraro, F., & Tabatabaei, M. [6] |
| 7 | Impact of the COVID-19 pandemic on clean fuel programmes in India and ensuring sustainability for household energy needs | The study of the COVID-19 pandemic impact on India's clean fuel programms found that the rural population to be unable to afford and access clean cooking fuels during the lockdown, and thus vulnerable to reversion to their traditional cooking methods using solid biomass fuels. The domestic air pollution caused by the use of polluting fuels was found to increase rural susceptibility to non-communicable diseases, and to intensify the risk and severity of COVID-19 infection. | It indicates the urgent need to expand sustainable energy solutions worldwide. | Ravindra, K., Kaur-Sidhu, M., Mor, S., Chakma, J., & Pillarisetti, A. [7] |
| 8 | Challenges and strategies for effective plastic waste management during and post COVID-19 pandemic | This study examined the negative impact of the current inadequate and inefficient waste management system to deal with the increased demand on plastic on the SDGs clean water, and calls for scientific sterilization and safe disposal of plastic wastes, as well as transition to using environmentally friendly materials. | It contributes to our attention to the needs of preventing falling from the current pandemic to another environmental and health crises, as well as the requirememts for increased investments, new technologies and infrastrutures for SDGs. | Vanapalli, K. R., Sharma, H. B., Ranjan, V. P., Samal, B., Bhattacharya, J., Dubey, B. K., & Goel, S. [8] |
| 9 | Covid-19 and Optimal Portfolio Selection for Investment in Sustainable Development Goals | This study addressed the reduced competitiveness of renewable energy in the face of reduced global energy demand and collapsed fossil fuel price, and proposes taxing pollution and waste such as CO2, NOx, and plastics, globally with the same tax rate. | The proposed global taxation on pollution can contribute to the desired portfolio allocation of assets needed for the implementation of SDG 7. | Yoshino, N., Taghizadeh-Hesary, F., & Otsuka, M. [9] |
| 10 | The impact of the COVID-19 pandemic on waste-to-energy and waste-to-material industry in China | This study examined the negative impact of the COVID-19 pandemic on achieving the SDGs in waste management in China. The annual revenues of solid waste industry reduced by 22%, the waste to material industry reduced by 28%, the waste disposal industry dropped by 10%, and the trade prices of secondary materials were only 43.4%-85.8% of the prices in the previous three years. | The findings of this study suggest the needs of a quantitative simulation of the long-term impact of COVID-19 pandemic, increased investments in and incentives on waste recycling, and an internal circulation system for waste management. | Zhou, C., Yang, G., Ma, S., Liu, Y., & Zhao, Z. [10] |
| 11 | Unleashing the convergence amid digitalization and sustainability towards pursuing the Sustainable Development Goals (SDGs): A holistic review | It identified the needs for categorization of the main SDGs research gaps; critical exploration of the potential contribution of digital paradigms, particularly Big Data and Artificial Intelligence, to overcome the identified caveats and pursuing the 2030 Agenda. | The findings might contribute to guiding and stimulating further research and science-policy dialogue on the promising nexus amid the SDGs and digitalization. | Del Río Castro, G., González Fernández, M. C., & Uruburu Colsa, Á. [11] |
| 12 | A hover view over effectual approaches on pandemic management for sustainable cities – The endowment of prospective technologies with revitalization strategies | The study aims to elucidate the pandemic characteristics in line with various temporal phases and its associated measures that proved effective in controlling the pandemic. In particular, it explores the role of technologies in seamless connectivity, rapid communication, mobility, technological influence in healthcare, digitalization influence, surveillance and security, Artificial Intelligence (AI), and Internet of Things (IoT). | The framing of insightful scenarios around the digitalized energy sector, an enhanced supply chain system with effective customer-retailer relationships to support the city during the pandemic scenario, and an advanced tracking system for containing virus spread, as well as revitalization strategies plays an important role in both recovering and nurturing sustainable development. | Elavarasan, R. M., Pugazhendhi, R., Shafiullah, G. M., Irfan, M., & Anvari-Moghaddam, A. [12] |
| 13 | Resilient regional food supply chains and rethinking the way forward: Key takeaways from the COVID-19 pandemic | This study found although vertically-integrated food supply chains (FSCs) can leverage large-scale production, streamline operations, and centralize planning and control to provide consumers with a consistent supply of food, they were seriously disrupted in the COVID-19 pandemic. However, regional food supply chains performed much better. | This study indicates that the regional food supply chains are more flexible that national and international ones to meet the communities needs in a major disruption like COVID-19. | Marusak, A., Sadeghiamirshahidi, N., Krejci, C. C., Mittal, A., Beckwith, S., Cantu, J., Morris, M., & Grimm, J. [13] |
